# Review of Deep Learning Approaches for the Segmentation of Multiple Sclerosis Lesions on Brain MRI

**DOI:** 10.3389/fninf.2020.610967

**Published:** 2020-11-20

**Authors:** Chenyi Zeng, Lin Gu, Zhenzhong Liu, Shen Zhao

**Affiliations:** ^1^School of Intelligent Systems Engineering, Sun Yat-Sen University, Guangzhou, China; ^2^RIKEN AIP, Tokyo, Japan; ^3^The University of Tokyo, Tokyo, Japan; ^4^Tianjin Key Laboratory for Advanced Mechatronic System Design and Intelligent Control, School of Mechanical Engineering, Tianjin University of Technology, Tianjin, China; ^5^National Demonstration Center for Experimental Mechanical and Electrical Engineering Education, Tianjin University of Technology, Tianjin, China

**Keywords:** deep learning, multiple sclerosis, brain MRI, review, segmentation

## Abstract

In recent years, there have been multiple works of literature reviewing methods for automatically segmenting multiple sclerosis (MS) lesions. However, there is no literature systematically and individually review deep learning-based MS lesion segmentation methods. Although the previous review also included methods based on deep learning, there are some methods based on deep learning that they did not review. In addition, their review of deep learning methods did not go deep into the specific categories of Convolutional Neural Network (CNN). They only reviewed these methods in a generalized form, such as supervision strategy, input data handling strategy, etc. This paper presents a systematic review of the literature in automated multiple sclerosis lesion segmentation based on deep learning. Algorithms based on deep learning reviewed are classified into two categories through their CNN style, and their strengths and weaknesses will also be given through our investigation and analysis. We give a quantitative comparison of the methods reviewed through two metrics: Dice Similarity Coefficient (DSC) and Positive Predictive Value (PPV). Finally, the future direction of the application of deep learning in MS lesion segmentation will be discussed.

## 1. Introduction

Multiple sclerosis (MS) is a chronic, autoimmune, and demyelinating disease with great clinical significance that affects the central nervous system (CNS). MS is a chronic disease that changes the morphology and structure of the brain due to the harm to the myelin sheath (Zhao et al., 2018). More importantly, MS can cause disability in young adults (Lladó et al., 2012). MS is relatively common in Europe, New Zealand, the United States, and parts of Australia. It has a major impact on the quality of life of the patients and their families due to its pathological characteristics.

**Figure 1:**
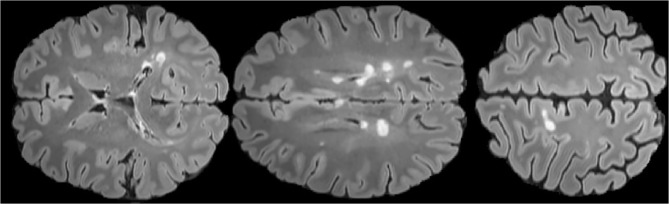
FLAIR axial MRIs of brain slices with MS lesions (white area) The figure comes from the public data set MICCAI2016 (Commowick et al., 2018).

The automatic segmentation of MS lesions through Magnetic Resonance Imaging (MRI) is of great clinical and engineering significance. Automatic segmentation of MS lesions is very important to help to detect diagnostic criteria for the disease which contains the spatial pattern of MS lesions in MRI (dissemination in space) and the emergence of new MS lesions(dissemination in time) (Polman et al., 2011). Besides, the automatic segmentation of MS lesions is essential for the quantitative analysis of the disease which is of great value in analyzing the progression of the disease and treatment options. Therefore, identifying and segmenting MS lesions is an indispensable step to characterize the disease and calculate and interpret more professional damage metrics. Before the emergence of the automatic segmentation of MS lesions, segmentation of the MS lesions were finished by experienced neuroradiologists. However, manual segmentation is a time-consuming and tedious process, and more importantly, it is poor efficiency due to intra-observer and inter-observer variability. Therefore, designing an excellent method for automatically segmenting MS lesions has great engineering significance. Figure 1 shows the morphology of MS lesions in MRI.

Although many methods for automatically segmenting MS lesions have been proposed in recent years, none of them are widely used in clinical practice. This is because this task still encounters many technical problems and challenges. The crucial difficulty is that the intensity distribution of MS and brain gray matter overlap in MRI (Sahraian and Radue, 2007). This is due to the limited resolution of the image, the heterogeneity of the lesion, and the complex shape of the brain tissue, which affects a large number of voxels located at the boundary of different tissues (Mortazavi et al., 2012). In addition, the variability of the appearance of the lesion and the magnetic resonance (MR) acquisitions are also a major challenge (Garćıa-Lorenzo et al., 2013). For example, MS lesions present hypointensities in T1-w MRI sequences, and hyperintensities in T2-w, Proton Density weighted(PD-w), and Fluid Attenuated Inversion Recovery T2(T2-FLAIR) MRI sequences, with respect to normal intensities (Hashemi et al., 2012). Due to these severe challenges, the performance of manual segmentation performed by experts outperforms automatic segmentation in most cases. Thus, there is still a demand for a better automatic segmentation method to be proposed to meet the requirements of clinical practice.

A comprehensive review is very important to help future generations design better automatic segmentation models based on the predecessors. In the past few years, there have also been related reviews (Danelakis et al., 2018; Kaur et al., 2020; Shanmuganathan et al., 2020) published. Danelakis et al. (2018) reviews the methods of automatically segmenting MS lesions and pointed out that MRI data acquisition and the injection of the contrast medium during data acquisition are great challenges in the future. Kaur et al. (2020) reviews the state-of-the-art methods by 2019 and lists the future directions obtained from these methods for future reference. Shanmuganathan et al. (2020) reviews the classification and segmentation methods of MS lesions and compares the classification and segmentation methods separately. Their comparison of various strategies shows that the segmentation methods based on deep learning achieve better performance.

Although the previous reviews (Danelakis et al., 2018; Kaur et al., 2020; Shanmuganathan et al., 2020) have done a great job, there are still no reviews that give a comprehensive overview of the deep learning-based automatic segmentation methods individually which achieve excellent performance. Although the previous review also included methods based on deep learning, there are some methods based on deep learning that they did not review. In addition, their review of deep learning methods did not go deep into the specific categories of CNN. They only reviewed these methods in a generalized form, such as supervision strategy, input data handling strategy, etc. In this paper, we focus on reviewing the deep learning-based MS lesion segmentation methods. Compared to previous reviews that categorize these methods based on supervision strategy, we divided these methods into two categories according to their CNN style: patch-wise segmentation and semantic-wise segmentation. The strengths and weaknesses of these two classifications are also given through our investigation and analysis. The fundamental goal of this survey is to help determine the most promising research direction of deep learning in this field.

The rest of this review is organized as follows: In section 2, public datasets and metrics for evaluating algorithm performance will be elucidated. Section 3 reviews various segmentation methods by classifying them into two different categories and presents a qualitative comparison of the algorithms reviewed. A discussion of the future directions is given in section 4.

## 2. Datasets and Metrics

In this section, we will introduce the datasets and metrics used by the methods we reviewed.

### 2.1. Datasets

The public datasets used by the deep learning-based MS lesion segmentation method has three: MICCAI 2008 (Styner et al., 2008), MICCAI 2016 (Commowick et al., 2018), ISBI 2015 (Carass et al., 2017). In Table 1, we illustrate these three public datasets.

**Table 1:** Public datasets used by the deep learning-based MS lesion segmentation.

**Dataset**	**Num of subjects**	**Training set:Test set**	**MRI sequence**	**MRI scan**
MICCAI 2008 (Styner et al., 2008)	45	20:25	T1-w, T2-w FLARE	3T Siemens Allegra 3T Siemens
MICCAI 2016 (Commowick et al., 2018)	53	15:38	T1-w T2-w PD-w T1-w Gd FLARE	Siemens Aera 1.5T Siemens Verio 3T Philips Ingenia 3T General Electric Discovery 3T
ISBI 2015 (Carass et al., 2017)	19	5:14	T1-w, T2-w FLARE, PD-w	3T Philips

### 2.2. Metrics

There are many evaluation measures used in the literature to quantify the performance of their methods. These evaluation measures are generally obtained by comparing the results of automatic segmentation with ground truth, and most of them are calculated by four basic terms (Goldberg-Zimring et al., 1998):

- TP(True Positive): The prediction is the MS lesion area, and the prediction is correct.
- TN(True Negative): The prediction is not the MS lesion area, and the prediction is not correct.
- FP(False Positive): The prediction is the MS lesion area, and the prediction is not correct.
- FN(False Negative): The prediction is not the MS lesion area, and the prediction is not correct.

We list the commonly used metrics in Table 2. In this review, we use DSC and PPV to compare various methods.

**Table 2:** Metrics for the reviewed methods.

**Metrics**	**Calculation**	**Substitute name**
Sensitivity (SEN) (Goldberg-Zimring et al., 1998)	$SEN=\frac{TP}{TP+FN}$	True positive rate
Specificity (SPE) (Goldberg-Zimring et al., 1998)	$SPE=\frac{TN}{TN+FP}$	True negative rate
Accuracy (ACC) (Wu et al., 2006)	$ACC=\frac{TN+TP}{TN+FP+TP+FN}$	
Dice similarity coefficient (DSC) (Dice, 1945)	$DSC=\frac{2TP}{FP+2TP+FN}$	*F*_1_ Score
Positive predictive value (PPV) (Altman and Bland, 1994)	$PPV=\frac{TP}{TP+FP}$	Precision
Fallout (FALL) (Udupa et al., 2006)	$FALL=\frac{FP}{TN+FP}$	False positive rate

## 3. Method

In this section, we first discuss the classification methods of all algorithms, and then we review the MS lesion segmentation methods based on deep learning and analyze their strengths and weaknesses according to the categorizations. Finally, we make a quantitative comparison of the methods we reviewed.

### 3.1. Categorization

The MS lesion segmentation task can be regarded as a semantic segmentation task, and each pixel (or voxel in 3D) of the input image needs to be classified as lesion or non-lesion. The methods we reviewed fall into two categories: patch-wise segmentation, semantic-wise segmentation. Patch-wise segmentation trains a CNN classifier to use the information of the pixel-centered patch to classify the pixel into two categories (lesions or non-lesions). Semantic segmentation trains a fully convolutional network to directly predict the lesion mask of the input image, so as to classify each pixel of the input image in a single forward propagation.

**Patch-wise segmentation** is the simplest segmentation strategy used when deep learning is just beginning to be applied to the segmentation of MS lesions. The segmentation strategy takes the pixel as the center and extracts a small patch of size N × N as the classifier input, and then they use the classifier to traverse the entire image. This strategy can make better use of contextual information around pixels. For example, Valverde et al. (2016) extracts 15 ×15 × 15 patches around each voxel from the MRI as input and then processes the input through two 3D convolutional layers. Then it output the probability of two possible classes(lesion and not lesion) through a fully connected layer and a softmax layer. In the patch-wise segmentation, a large number of redundant calculations are caused by overlapping patches, which decreases the calculation efficiency greatly. **Semantic-wise segmentation** is first proposed by Brosch et al. (2015). The input of semantic-wise segmentation can be the entire MRI volume or a relatively large patch. In semantic-wise segmentation, there will be no redundant calculations caused by overlapping patches. In Brosch et al. (2015), it takes the entire MRI volumes as input. Then feed the input into the network consists of a convolutional layer (LeCun et al., 1998) and a deconvolution layer (Zeiler et al., 2011) to predict the lesion mask.

### 3.2. Patch-Wise Segmentation

Patch-wise segmentation potentially converges faster when training the model because it randomly samples the patches over the dataset (LeCun et al., 1998). Besides, it is easier to deal with the problem of class imbalance. However, the time required to train a complicated patch-wise method can make the method infeasible when the number and size of patches are large. In addition, it has a lot of redundant calculations due to patch overlap.

Yoo et al. (2014) uses deep learning for feature learning and random forest for classification. They first train a model on a large amount of unlabeled data to recognize common patterns and then add labels to the training subset so that both features and labels can be used for segmentation tasks. Vaidya et al. (2015) uses 3D patches as input, via 3D convolutional network to classify each patch into two categories. Havaei et al. (2016) proposed a CNN network that can segment images from systematic multi-modal datasets. The method maps the input image to an embedding space. In this embedding space, arithmetic calculations (such as computing moments of a collection of vectors) are well-defined and can be used for different modalities available during inference time. These calculated moments can be further used to predict the final segmentation. This algorithm improves the robustness against missing input data modalities. Method in Birenbaum and Greenspan (2016) takes patches from multiple images, multiple views and multiple time points as input. It can be divided into two stages. The first stage uses FLARE and white matter (WM) prior to calculating the candidate voxel, and the second stage uses multi-view CNN to predict lesion probability for each voxel in MRI. It is the first deep learning method that uses longitudinal data for segmentation. Valverde et al. (2017) proposed a cascade structure consisting of two stages to segment MS lesions. When training the model, they manually select the training data to solve the problem of imbalance between positive and negative samples. The first stage is used to output voxels with a large probability of being a lesion, and the second stage further infers whether the voxels output by the first stage are lesions, and finally via set threshold to get binary output masks. On the basis of the previous work, Valverde et al. (2017, 2019) studied the influence of intensity domain adaptation on model performance. Alshayeji et al. (2018) proposed an effective method to simplify the pre-processing steps and reduce the processing time using heterogeneous single-channel MRI. They extract the features of the lesion use mathematical operations and morphological operations, and train an Multilayer Perceptron (MLP) for classification to reduce processing time. Essa et al. (2020) performs patch-wise R-CNN on the input image of each modality to generate a probabilistic output of locations of MS lesion, they input the extracted patches as the proposed regions into the RCNN output probabilistic output of lesion existence. They propose an adaptive neuro-fuzzy inference system to show how different MRI modalities are integrated, and they use this system to fuse the output of each MRI modality to get the final segmentation result.

### 3.3. Semantic-Wise Segmentation

Compared with patch-wise segmentation, semantic-wise segmentation requires only one forward propagation to classify all pixels of the input image and it has higher computational efficiency. But for the task of MS lesion segmentation, semantic-wise segmentation is prone to overfitting during training due to class imbalance, because in the MRI of MS lesions, the area of the lesion area is much smaller than the non-lesion area.

Brosch et al. (2016) combines the advantages of Brosch et al. (2015) and U-net (Ronneberger et al., 2015). It contains two paths: one is an encoding path composed of convolutional layers and pooling layers, and the other is a decoding path composed of deconvolutional layers and unpooling layers. A shortcut connection is built between the two paths. Compared with U-net (Ronneberger et al., 2015), it uses a deconvolution layer instead of upsampling, so there is no need to specially process the boundary regions. McKinley et al. (2016) introduces the nabla net, which combines the low-level features learned by the fully convolutional network and the high-level features learned by the encoder-decoder network to output a probability map. Zhang et al. (2018) uses LinkNet (Chaurasia and Culurciello, 2017) as their base segmentation network. LinkNet is an encoder-decoder network with an additional link between encoder and decoder. They add a loss function related to classification to the conditional generative adversarial network (cGAN) to achieve semantic segmentation more efficiently. Roy et al. (2018) uses parallel pathways to process different MRI image patches, and then concatenate the outputs of these pathways to predict the membership function of the patch through another convolution filter. It does not have a fully connected layer, it replaces the fully connected layer with a fully convolutional layer to get less false positives. In order to solve the problem of MS lesions have huge variability in size and DSC is not differentiable which result in can not be used directly for gradient descent, Wang et al. (2018) segments large and small lesions separately and propose a new activation function to facilitate network training. Aslani et al. (2018)uses 2D slices as input and a 2D encoder-decoder network to segment MS lesions to avoid the problems like the oversight of global information of patch-wise methods and the overfitting of 3D segmentation due to the problem of class imbalance. Kumar et al. (2018) combines the advantages of SegNet (Badrinarayanan et al., 2017) and U-net (Ronneberger et al., 2015) that U-net captures multi-scale information more effectively and SegNet has fewer parameters and faster training. Aslani et al. (2019) focuses on whole-brain slice-based segmentation in order to prevent the overfitting problem of 3D-based segmentation and the problem that patch-based segmentation cannot use global information. In addition, it also uses multi-level feature fusion to better use contextual information for segmentation. Zhang et al. (2019a) uses fully convolutional densely connected networks (Jégou et al., 2017) for MS lesion segmentation, and uses 2.5D stacked slices as input to improve segmentation performance. The term 2.5D is defined as slices stacked along three orthogonal planes (axial, sagittal, and coronal). Zhang et al. (2019b) proposes a recurrent slice-wise attention network by repeatedly using the contextual information of MS lesions to respond to the problem that Recurrent Neural Network (RNN) and long short-term memory (LSTM) have inherent flaws to capture long-term dependencies. Aslani et al. (2020) proposed a regularized network with an auxiliary loss function which makes the model ignore domain-specific information to handle the problem of domain shift. Gessert et al. (2020a) proposes a 4D deep learning network to improve the activity segmentation performance of MS lesions. It adds a 3D volume of historical time point to the input of the network and designs a new multi-encoder-decoder architecture that uses convolutional-recurrent units for time aggregation. In addition, they also explored whether adding an additional past time point to the input can improve segmentation performance. La Rosa et al. (2020) uses magnetization-prepared 2 rapid acquisition with gradient echo(MP2RAGE) MRI to segment cortical lesions(CLs). Compared to this method, other methods just segment white matter lesions(WMLs). Its network structure is based on 3D U-net (Çiçek et al., 2016). For the domain shift problem in MS lesion segmentation, Ackaouy et al. (2020) proposes an unsupervised method that learns a shared representation of the source and target domains. Gessert et al. (2020b) segment the newly emerging MS lesions by attention mechanism with two paths network while the general method only considers MS lesions segmentation in a single MRI volume. This task is particularly challenging because new lesions are minute, changes are subtle. Gabr et al. (2020) study how deep learning based on full convolutional neural networks (FCNN) performs when there is more data. They train, verify, and test on a dataset containing 1,000 MRI and got great results(DSC:0.95). Coronado et al. (2020) evaluates the performance of deep learning in segmenting gadolinium-enhancing lesions using a large cohort of MS patients.

### 3.4. Quantitative Comparation

In this subsection, reviewed MS lesion segmentation methods based on deep learning will be compared. Table 3 shows the main performance comparison of various methods. Each method is analyzed through the dataset used, the input data dimension, the CNN style, and the performance (DSC Dice, 1945, PPV Altman and Bland, 1994).

**Table 3:** Comparison of reviewed methods.

**Methods**	**Database**	**Dim**	**CNN style**	**DSC**	**PPV**
Roy et al. (2018)	ISBI 2015	3D	Semantic-wise	0.524	0.866
Birenbaum and Greenspan (2016)	ISBI 2015	3D	Patch-wise	0.627	0.789
Valverde et al. (2019)	ISBI 2015	3D	Patch-wise	0.63	0.840
Aslani et al. (2019)	ISBI 2015	2D	Semantic-wise	0.61	0.899
Aslani et al. (2018)	ISBI 2015	2D	Semantic-wise	0.698	0.74
Zhang et al. (2019a)	ISBI 2015	2.5D	Semantic-wise	0.693	0.908
Havaei et al. (2016)	MICCAI 2008	2D	Patch-wise	0.832	N/A
Valverde et al. (2017)	MICCAI 2008	3D	Patch-wise	0.871	0.786
Brosch et al. (2016)	MICCAI 2008	3D	Semantic-wise	0.840	N/A
Valverde et al. (2016)	MICCAI 2016	3D	Patch-wise	0.541	N/A
McKinley et al. (2016)	MICCAI 2016	3D	Semantic-wise	0.591	N/A
Kazancli et al. (2018)	Proprietary	3D	Patch-wise	0.575	N/A
La Rosa et al. (2020)	Proprietary	3D	Semantic-wise	0.60	0.64
Brosch et al. (2015)	Proprietary	3D	Semantic-wise	0.355	0.414
Gabr et al. (2020)	Proprietary	3D	Semantic-wise	0.95	N/A
Coronado et al. (2020)	Proprietary	3D	Semantic-wise	0.77	N/A
Zhang et al. (2018)	Proprietary	2D	Semantic-wise	0.672	0.724
Aslani et al. (2020)	Proprietary	3D	Semantic-wise	0.50	0.519
Gessert et al. (2020a)	Proprietary	4D	Semantic-wise	0.64	N/A
Gessert et al. (2020b)	Proprietary	3D	Semantic-wise	0.656	N/A
Zhang et al. (2019b)	Proprietary	2D	Semantic-wise	0.660	N/A

It can be seen from Table 3 that there are many methods that still use their private datasets which is not convenient to compare the performance of the methods quantitatively.

## 4. Future Direction

Although deep learning has achieved great performance in MS lesion segmentation tasks compared with traditional methods (Danelakis et al., 2018), there are still some problems that limit the potential of deep learning in this field: dataset scale, data imbalance, domain shift. Deep learning has also achieved great performance in other fields of medical images (Zhao et al., 2017, 2019a,b; Xu et al., 2018). We believe that borrowing deep learning methods from other fields into MS automatic segmentation can help design better segmentation methods. In the later part of this section, we will discuss some possible solutions to these problems as well as some new research problems in this field.

Transfer learning can be a future direction to deal with the problem of small data sets. This problem not only exists in the study of MS lesion segmentation, but it is also a coexisting problem in medical image processing due to the difficulty of medical image acquisition and labeling. To solve the problem of a small dataset scale, it can be achieved through transfer learning (Pan et al., 2011). Transfer learning is a learning method for small datasets. First, a deep learning network with great performance is trained on a large dataset, and then the network is fine-tuned on a smaller dataset for specific problems. For example, Shin et al. (2016) reported that they performed transfer learning from pre-trained models on the ImageNet dataset and then fine-tuned on lymph node and interstitial lung diseases instead of training from scratch to achieve great performance. But we believe that directly transferring from natural images to medical images may not be the best transfer learning solution, because natural images and medical images are very different. We think it is possible to perform transfer learning from large medical image data sets, such as DeepLesion (Yan et al., 2018).

Designing a specific loss function may be a direction to solve the problem of data imbalance in the future. The pattern of manifestation of data imbalance in MS lesion segmentation is class imbalance. In a single MRI volume, the number of voxels with lesions is much smaller than the number of voxels without lesions, which will bring problems such as overfitting to the network training (Li et al., 2019). The impact of this problem on the patch-wise CNN style is less than that of the semantic-wise CNN style because the patch-wise CNN style classifies each voxel separately. In the patch-wise CNN style, the ratio of positive samples and negative samples of training data can be adjusted artificially to balance the class (Valverde et al., 2017). However, for the semantic-wise CNN style, all voxels are classified in a forward propagation, which makes it difficult to artificially adjust the ratio of positive and negative samples in the training data. Therefore, the class imbalance problem of the semantic-wise CNN style requires to be solved from another aspect. Through our investigation of imbalance problems in other fields, we found that the current mainstream method to solve this problem is to design a loss function carefully (e.g., Sudre et al., 2017; Wong et al., 2018; Kervadec et al., 2019; Li et al., 2019). They have achieved great results in other segmentation tasks such as ischemic stroke injury by proposing a loss function for class imbalance. We believe that this will also help reduce the impact of overfitting caused by the data imbalance in the segmentation of MS lesions.

Collaborative image and feature adaptation can improve the performance of the domain adaptive model to a certain extent. The domain shift refers to the problem that the model performs well on the source domain, but performs much worse on the target domain. Although there are some methods (Valverde et al., 2019; Ackaouy et al., 2020) in MS lesion segmentation that proposes domain adaptation models to solve the problem of domain shift, they only implement domain adaptation from the perspective of feature adaptation. Chen et al. (2019) proposes a domain adaptive method from two perspectives of image and feature, and verified their method on a cross-modal heart structure segmentation challenge. They choose the source domain as MRI modal data and the target domain as computed tomography(CT) modal data. They restored the performance degradation from 17.2 to 73.0%. We think that improving the performance of the domain adaptive model from both image and feature aspects is a future direction in MS segmentation.

Through our research, we found that some recent work began to introducing the sequence model (Gessert et al., 2020a,b) to segment the activity of MS lesions. The task of segmentation of multiple sclerosis lesion activity is to detect the appearance of new and enlarged lesions between the baseline and subsequent brain MRI scans (Gessert et al., 2020b). We think this is also a future direction for the segmentation task. More sequence models can be used to analyze the improvement and deterioration of patient lesions (e.g., LSTM). Cai et al. (2017) applies contextual LSTM (CLSTM) to the output layer of deep CNN and achieves sharper pancreas segmentation by capturing the context information of adjacent slices. Spatiotemporal regularization (Zheng et al., 2019) may improve the performance of activity segmentation and structure tensor (Zheng et al., 2020) can help more accurately capture the changes in the edge of the lesion.

## 5. Conclusion

In this review, we have done a detailed survey on the method of MS lesion segmentation based on deep learning, and we reviewed the commonly used public datasets and evaluation metrics of this segmentation task. We categorize these methods according to the CNN style they use. It is difficult to compare various methods because the datasets they use are not only public datasets but also their own proprietary data sets. We use DSC and PPV for quantitative comparisons including those that use proprietary datasets. The future direction and some potential problems are also illustrated.

Although deep learning greatly improves the performance of automatic segmentation methods, it is still challenging to directly use in clinical analysis. Collecting large-scale data sets to tap the potential of deep learning can help accelerate its application in clinical medicine, and there is still a lot of room for improvement for deep learning-based methods. The automatic segmentation method with better performance and stronger robustness is undoubtedly beneficial to the doctor's pre-diagnosis and post-treatment of the patient's condition.

## Data Availability Statement

Publicly available datasets were analyzed in this study. This data can be found here: http://www.ia.unc.edu/MSseg/; https://smart-stats-tools.org/lesion-challenge-2015; https://portal.fli-iam.irisa.fr/msseg-challenge/overview.

## Author Contributions

CZ was responsible for writing the paper. ZL was responsible for consulting the literature. LG was responsible for method comparison. SZ was responsible for revising the article. All authors contributed to the article and approved the submitted version.

## Conflict of Interest

The authors declare that the research was conducted in the absence of any commercial or financial relationships that could be construed as a potential conflict of interest.
